# Fattening by Dietary Replacement with Fly Maggot Larvae (*Musca domestica*) Enhances the Edible Yield, Antioxidant Capability, Nutritional and Taste Quality of Adult Chinese Mitten Crab *Eriocheir sinensis*

**DOI:** 10.3390/foods14071250

**Published:** 2025-04-03

**Authors:** Xiao Liang, Changle Qi, Jinyu Tang, Ting Ye, Bao Lou, Fuyong Huang

**Affiliations:** 1 State Key Laboratory for Quality and Safety of Agro-Products, Institute of Hydrobiology, Zhejiang Academy of Agricultural Sciences, Desheng Middle Road 298, Hangzhou 310021, China; liangx@zaas.ac.cn (X.L.); tangjy@zaas.ac.cn (J.T.); yet@zaas.ac.cn (T.Y.); loubao@zaas.ac.cn (B.L.); 2 National-Local Joint Engineering Laboratory of Aquatic Animal Genetic Breeding and Nutrition, Zhejiang Provincial Key Laboratory of Aquatic Resources Conservation and Development, College of Life Science, Huzhou University, Huzhou 313000, China; qichangle1989@163.com

**Keywords:** housefly maggot larvae, *Eriocheir sinensis*, fattening, textural properties, flavor characteristics

## Abstract

Housefly maggot larvae (HML) have been identified as a potential alternative animal diet for the fattening process of the Chinese mitten crab (*Eriocheir sinensis*). However, the feasibility and potential impacts of HML supplementation require further investigation. The present study evaluated the effects of dietary HML on the growth indices, nutrient compositions, antioxidant activity, and texture profiles of edible tissues of *E. sinensis*. The results showed that dietary HML supplementation effectively improved the hepatic steatosis index of both genders and sweet amino acid content of edible tissues (except for male gonad) (*p* < 0.05). Additionally, dietary HML significantly increased the total antioxidant capacity in the gonad and female muscle (*p* < 0.05). For the textural properties, HML feeding significantly improved the adhesiveness in the male muscle, and the cohesiveness, chewiness, and resilience in the female muscle (*p* < 0.05). Furthermore, HML feeding significantly decreased the levels of the equivalent umami concentration of the male gonad, male muscle, and hepatopancreas (*p* < 0.05). Conversely, HML feeding significantly increased the sweetness value in the muscle, hepatopancreas, and female gonad (*p* < 0.05). Our findings indicate that HML could serve as a viable alternative feed for fattening to improve the edible yield and change the flavor characteristics in *E. sinensis.*

## 1. Introduction

The Chinese mitten crab (*Eriocheir sinensis*) is an important aquaculture species and highly prized by consumers in China due to its high nutritional value and unique flavor [1,2]. The total production of *E. sinensis* in China ranks first globally, with an annual yield nearing 1 million tons [3]. Prior to market harvest in September, the fattening of adult *E. sinensis* is typically required, as it directly enhances the overall quality and yield of crabs, thus ensuring better market prices [4,5]. The duration of the fattening period and efficient dietary feed are crucial factors influencing both nutrition deposition and gonad development in crabs [5,6]. Previous research suggests that the optimal fattening period for pond-reared *E. sinensis* ranges from 40 to 60 days [4,6]. Common dietary options for fattening include iced trash fish (IF), soybean, cornmeal, and formulated diets (FDs) [5]. IF, once considered the “standard food” for crab fattening, has been associated with several issues, including source instability, unpredictable quality, nutritional imbalances, and water pollution [7]. As a result, numerous studies have focused on developing high-quality FDs or alternative protein sources to enhance fattening practices [5,8].

The crude protein level in most insects ranges from 40% to 63%, which have been recognized as stable sources of dietary proteins for aquaculture [8,9,10,11]. Housefly maggots (*Musca domestica*; HM) are promising and highly nutritious resources, offering significant potential as a protein source for crabs [11,12,13]. The crude protein content of HM ranges from 43% to 62% [14], and HM are rich in monounsaturated fatty acids, B-complex vitamins, phosphorus, and trace elements [13,15]. Previous studies have demonstrated that insect meal can effectively replace fish meal in aquaculture diets without adversely affecting the growth of various fish species, including barramundi (*Lates calcarifera*), gilthead seabream (*Sparus aurata*), and European sea bass (*Dicentrarchus labrax* L.) [16,17,18]. Moreover, incorporating HM larvae (HML) in fish diets has the potential to improve growth rates and feed efficiency without inducing physiological stress [11,19]. Dietary HML supplementation has been shown to improve the hardness and moisture in fish muscles, enhancing the flesh quality of Nile tilapia (*Oreochromis niloticus*) [20]. Additionally, HML-supplemented diets promote the growth of swamp eel (*Monopterus albus*) and support gonadal development in *Clarias gariepinus* (Burchell, 1822) [21,22]. In crustaceans, replacing fish meal with defatted insect meal, such as yellow mealworm (*Tenebrio molitor*), significantly increased the growth and survival rates of juvenile Pacific white shrimp (*Litopenaeus vannanmei*) [23], while dietary supplementation with black soldier fly (*Hermetia illucens*) enhanced the immunity, gut microbiota, and protein content in freshwater crayfish marron (*Cherax cainii*) [24]. Furthermore, the exoskeleton of insects contains chitin, a structural polysaccharide that may contribute to the formation of the exoskeletons in crustaceans [25]. However, studies on the effects of HML on the growth and health of crustaceans remain limited.

Although preliminary investigations have revealed the regulatory effects of HML on growth and nutrient metabolism in decapod crustaceans [12,19], their efficacy in enhancing nutritional quality remains insufficiently explored. Therefore, this study aimed to systematically evaluate the impacts of HML supplementation on the growth indices and nutrient composition of edible tissues in *E. sinensis* during the fattening period. Specifically, we assessed various growth parameters, the antioxidant status, amino acid (AA) and nucleotide composition, texture profiles, and flavor characteristics of different edible tissues. The findings will validate its application potential as a functional feed additive and establish a theoretical foundation for precision nutrient regulation in crustacean aquaculture.

## 2. Materials and Methods

### 2.1. Experimental Setup and Culture Management

This study was conducted in Changxing Town, Huzhou, Zhejiang Province (China). Healthy, active, and intact *E. sinensis* were randomly selected from a local farm in September. A total of approximately 240 female and 240 male crabs were stocked into two separate outdoor earthen ponds. Each pond was subdivided into six parallel sections (length × width × depth = 10 m × 8 m × 1.2 m) using purse seines, with a stocking density of 40 crabs per section. Each pond contained two experimental groups, with three sections designated per group. The initial body weights of the adult female and male crabs, post-puberty molt, were 150 ± 17 g and 200 ± 19 g, respectively. Based on a previous study [6], this experiment commenced on 5 September 2023, and lasted for 40 days. During the fattening period, water quality parameters, including dissolved oxygen (>5 mg/L), ammonia nitrogen (<0.3 mg/L), nitrite (<0.01 mg/L), and pH (7.5–9.0), were maintained within normal reference ranges. *Elodea canadensis* was transplanted to cover more than 60% of the area in each region, providing shelter for the crabs and assisting in the maintenance of water quality.

**Figure 1:**
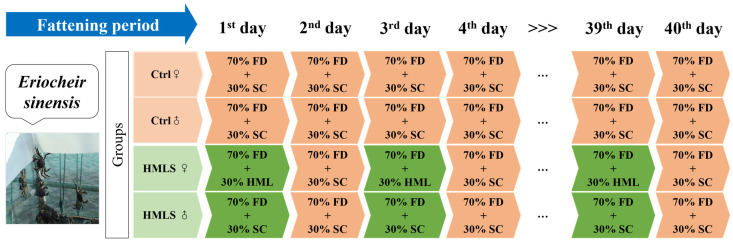
General experimental design of the feeding trial. Note: This feeding trial started on September 5th in 2023 and lasted for 40 days. Experimental groups consisted of control (Ctrl) group and HML supplementation (HMLS) group. FD: formula diets; SC: soybean and corn combinations.

In this study, both the component percentage in the fattening diet combinations and administration protocols were designed in accordance with practical aquaculture production practices. The current aquaculture operations commonly employ formula diets supplemented with plant-based ingredients (soybean, corn, etc.) as standard practice [5]. Consequently, the composition of the fattening diets is shown in Figure 1, and the nutritional composition of HML is shown in Supplementary Table S1. The crabs were fed once daily, with the feeding amount comprising approximately 2–4% of their body weight, at 17:00. In the HML supplementation (HMLS) group, the crabs were provided with formula diets and HML on the first day, followed by non-HML supplementation diets (a combination of formula diets, soybean, and corn) on the second day, and this alternated daily thereafter. The control group received non-HML supplementation diets continuously. The feeding amount was adjusted based on residual feed, temperature, and water quality.

### 2.2. Sample Collection and Processing

At the end of the experiment, the crabs were fasted for 24 h prior to sampling. Referring to classical studies on sample size selection [4,6], eighteen crabs per group (six individuals from each section) were randomly selected, then dissected. The meat, gonad, and hepatopancreas were weighed to calculate the meat yield (MY), hepatosomatic index (HSI), and gonadosomatic index (GSI). Nine female and nine male crabs were randomly selected for the measurement of antioxidation capacity and astaxanthin contents. For AA composition and nucleotide analysis, another set of nine female and nine male crabs were selected, gently blotted with a towel to remove surface moisture, and their edible tissues were carefully removed from the carapace cavity and body parts of the freshly uncooked crabs. All edible parts were steamed for 15 min over boiling water, after which the homogenates were stored for later analysis. All samples were stored separately at −80 °C. Claw muscle samples were obtained, naturally cooled to room temperature, and subsequently analyzed for texture.

The MY, HSI, GSI, and total edible yield (TEY) of the crabs were calculated using the following formulas:

MY (%) = 100 × Meat wet weight/Body wet weight.

HSI (%) = 100 × Hepatopancreas wet weight/Body wet weight.

GSI (%) = 100 × Gonad system wet weight/Body wet weight.

TEY (%) = MY + HSI + GSI.

### 2.3. Antioxidant Activity Analysis

The activities of superoxide dismutase (SOD) and total antioxidant capacity (T-AOC), and the malonaldehyde (MDA) content in the muscle, gonad, and hepatopancreas tissues were measured using specific commercial kits (Nanjing Jiancheng Biotech Co., Ltd., Nanjing, China), following the manufacturer’s protocols. The SOD was determined at 550 nm by the hydroxylamine method. The T-AOC was determined at 405 nm by the 2,2′-amino-di (2-ethylbenzothiazoline sulfonic acid-6) ammonium salt (ABTS) method. The content of MDA was determined at 532 nm by the thiobarbituric acid (TBA) method. The total protein content in tissue samples was measured at 595 nm by the Coomassie brilliant blue method to calculate the above indicators. Three replicates for each sample were analyzed.

### 2.4. Determination of Astaxanthin Content

Tissues from all individuals (n = 18) were sampled, stored in liquid nitrogen, and then transferred to −80 °C. According to the method of Su et al. [26], astaxanthin content was determined using a high-performance liquid chromatography (HPLC) system (Shimadzu, LC20AD, Kyoto, Japan). Firstly, all samples were dried in a vacuum freeze dryer (Scan Vac Cool Safe, Labo Gene, Lynge, Denmark) and then ground into powder (under light protection). About 0.2 g of tissue powder was placed in a 10 mL centrifuge tube and extracted with 4 mL of acetone (analytical grade), shaken in the dark at 25 °C (200 rpm) for about 10. Then, the supernatant was filtered through a 0.20 µm filter membrane, and 20 µL of the supernatant was injected into the system. Separation was performed using a C30 column (4.6 mm × 250 mm, 3 µm) (Thermo Fisher Scientific, Waltham, MA, USA), with a mobile phase of n-hexane–acetone (83:17, *v*/*v*) at a flow rate of 1 mL/min and a column temperature of 25 °C. Detection of astaxanthin was carried out using a UV–Vis detector at a wavelength of 478 nm.

### 2.5. Texture Profile Analysis (TPA) of Muscles

To assess the TPA of muscle, nine individuals were randomly selected from three experimental cages (three crabs per cage), and the propodus muscle of the crab claw was dissected for TPA measurement. TPA was performed using a TA-XT Plus Micro TPA (Stable Micro Systems, Surrey, UK) equipped with a flat-bottomed cylindrical probe P/50. The sample collection method followed the protocol outlined in previous studies [27,28]. The propodus muscle samples were first heated and then allowed to naturally cool to room temperature. Each sample was then trimmed into cubes (16 mm diameter, 10 mm height), and the TPA of muscle was conducted immediately. The mean TPA values obtained from the two claws of each crab were used as the final result for that individual.

The Universal TA Texture Analyzer (Tengba, Shanghai, China) was employed for the analysis, measuring various texture attributes, including hardness, springiness, chewiness, gumminess, adhesiveness, cohesiveness, and resilience. A cylindrical probe with a diameter of 36 mm and a speed of 1 mm/s was used. The distance mode was selected with a movement distance of 1.5 mm. The contact induction force was set to 5 gf, and the interval between two depressions was 5 s. Two consecutive compression cycles were performed, with the deformation set to 50% of the sample’s initial height.

### 2.6. Free AA Determination

The free AAs in edible tissues were analyzed using the method described in a previous study [8].

### 2.7. Nucleotide Analysis

The nucleotides in edible tissues were analyzed according to the methodology outlined in a previous study [29]. The nucleotides responsible for eliciting the umami sensation include adenylate (AMP), guanylate (GMP), and inosinate (IMP).

### 2.8. Flavor Evaluation Indices

The threshold values of AAs and nucleotides were obtained from prior studies [30,31,32]. The taste activity value (TAV) was calculated as the ratio of the measured concentration of each AA or nucleotide to its respective threshold value. Compounds with a TAV greater than 1 were considered contributors to taste.

To assess the intensity of the umami flavor resulting from the synergistic effects of AAs and nucleotides, the equivalent umami concentration (EUC) was calculated as follows:

*EUC* = Ʃ *a_i_b_i_* + 1218 (Ʃ *a_i_b_i_*) (Ʃ *a_j_b_j_*),

a*_i_* is the concentration of umami amino acid (UAA) (Asp or Glu, g/100 g); b*_i_* is the ratio of the umami concentration of Asp or Glu to that of monosodium glutamate (MSG) (Glu, 1; Asp, 0.077); a*_j_* is the concentration of umami 5′-nucleotide (IMP, GMP, or AMP, g/100 g); b*_j_* is the ratio of the umami concentration of a 5′-nucleotide to that of IMP (IMP, 1; GMP, 2.3; AMP, 0.18); and 1218 is a synergistic constant based on the concentration of g/100 g used.

### 2.9. Statistical Analyses

Statistical analysis was conducted using SPSS 24.0 (IBM, New York, NY, USA) using one-way ANOVA. Data are presented as the mean ± standard error (SE). To assess differences between two groups, multiple unpaired *t*-tests were performed and validated by Turkey multiple comparison tests (Turkey HSD). A *p*-value of < 0.05 was considered statistically significant. Radar plots and correlation analysis among the variables assessed with Spearman’s correlation, as well as other graphs, were generated using OmicShare tools, a free online platform for data analysis and visualization (http://www.omicshare.com/tools, accessed on 30 April 2024).

## 3. Results

### 3.1. Growth Performance in Adult E. sinensis

**Figure 2:**
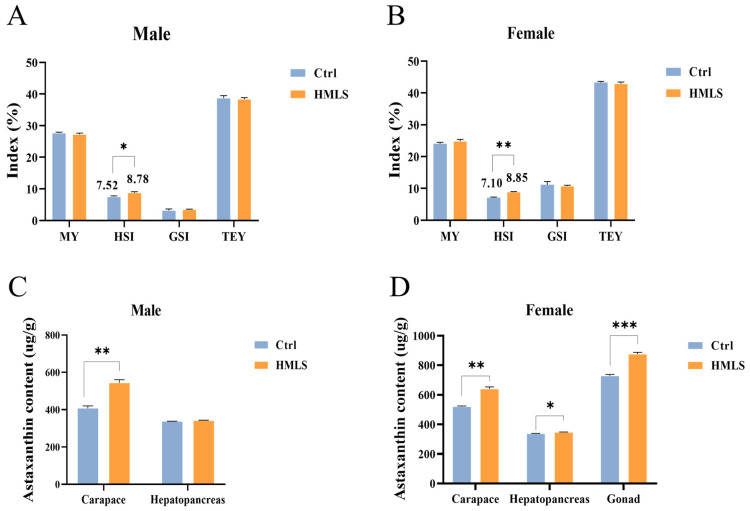
Effects of dietary HML on edible tissues and astaxanthin content in adult *E. sinensis.* (**A**,**B**) The results are presented as the means ± SE (n = 18). (**C**,**D**) The results are presented as the means ± SE (n = 9). An asterisk (*) indicates significance at the 0.05 level, ** at the 0.01 level, and *** at the 0.001 level.

After a 40-day fattening period, the HSI in both the male and female crabs of the HMLS group was significantly higher than that of the Ctrl group (*p* < 0.05). However, no significant differences were observed between the HMLS and Ctrl groups in terms of the MY, GSI, or TEY of crabs (Figure 2A,B).

### 3.2. Astaxanthin Content in Different Edible Tissues of E. sinensis

Astaxanthin content was measured in adult crabs from both the Ctrl group and HMLS group. In comparison to the Ctrl group, the HMLS group exhibited a significant increase in astaxanthin content in the carapace (*p* < 0.01). However, no significant differences were observed in the hepatopancreas between the Ctrl and HMLS groups (*p* > 0.05) (Figure 2C). Additionally, in female crabs, the astaxanthin content was significantly higher in the carapace (*p* < 0.01), hepatopancreas (*p* < 0.05), and gonad (*p* < 0.001) of the HMLS group compared to the Ctrl group (Figure 2D).

### 3.3. Antioxidant Capacity in Different Edible Tissues of E. sinensis

**Figure 3:**
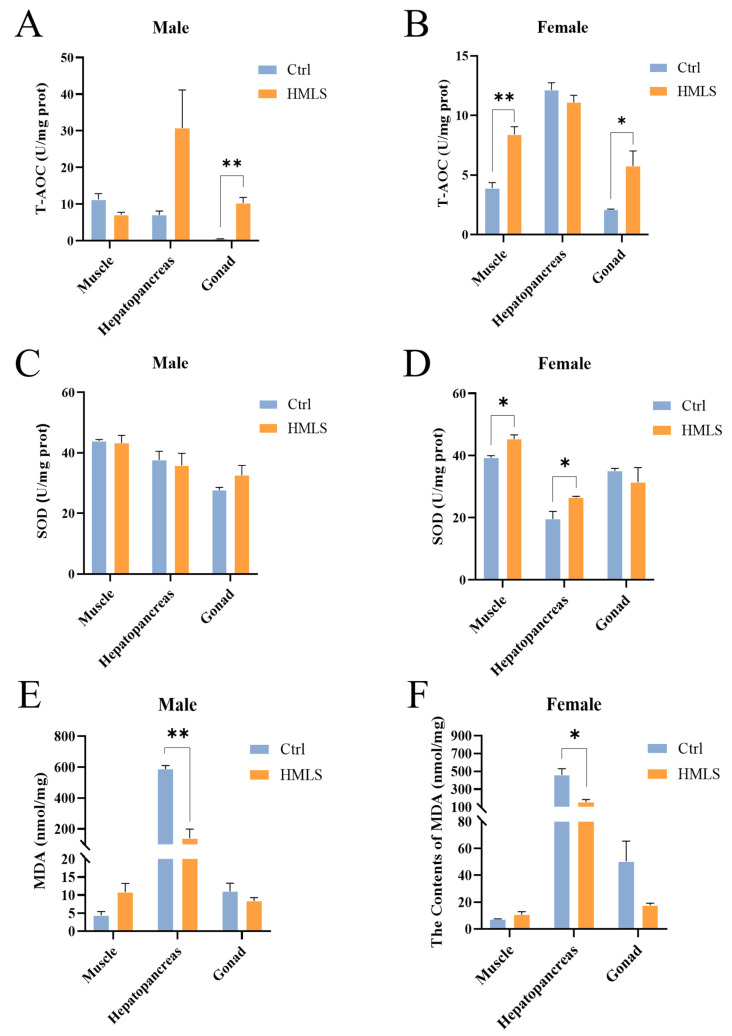
Effects of dietary HML on antioxidant parameters of edible tissues in adult *E. sinensis*. (**A**,**B**) T-AOC activity. (**C**,**D**) SOD activity. (**E**,**F**) MDA content. The results are presented as the means ± SE (n = 9). An asterisk (*) indicates significance at the 0.05 level and ** at the 0.01 level.

As illustrated in Figure 3A,B, dietary supplementation with HML significantly enhanced the T-AOC in the gonad (*p* < 0.05) and female muscle (*p* < 0.01) of *E. sinensis*. No significant differences were observed in the SOD activities of male edible tissues between the Ctrl group and HMLS group (Figure 3C). However, dietary HML significantly increased the SOD activities in the muscle and hepatopancreas of female crabs (*p* < 0.05) (Figure 3D). Furthermore, dietary HML significantly decreased the MDA content in the hepatopancreas of both sexes (*p* < 0.05) (Figure 3E,F).

### 3.4. Textural Properties

Texture profile analysis (TPA) was conducted to assess the impact of dietary HML on the textural characteristics of various edible tissues, including hardness, adhesiveness, springiness, cohesiveness, gumminess, chewiness, and resilience (Table 1). In comparison to the Ctrl group, adhesiveness in the male muscle was significantly increased in the HMLS group (*p* < 0.05). Additionally, dietary HML significantly increased the cohesiveness (*p* < 0.01), chewiness (*p* < 0.05), and resilience (*p* < 0.01) of the female muscle.

### 3.5. Free AA Profiles in Different Edible Tissues of Steamed E. sinensis

**Figure 4:**
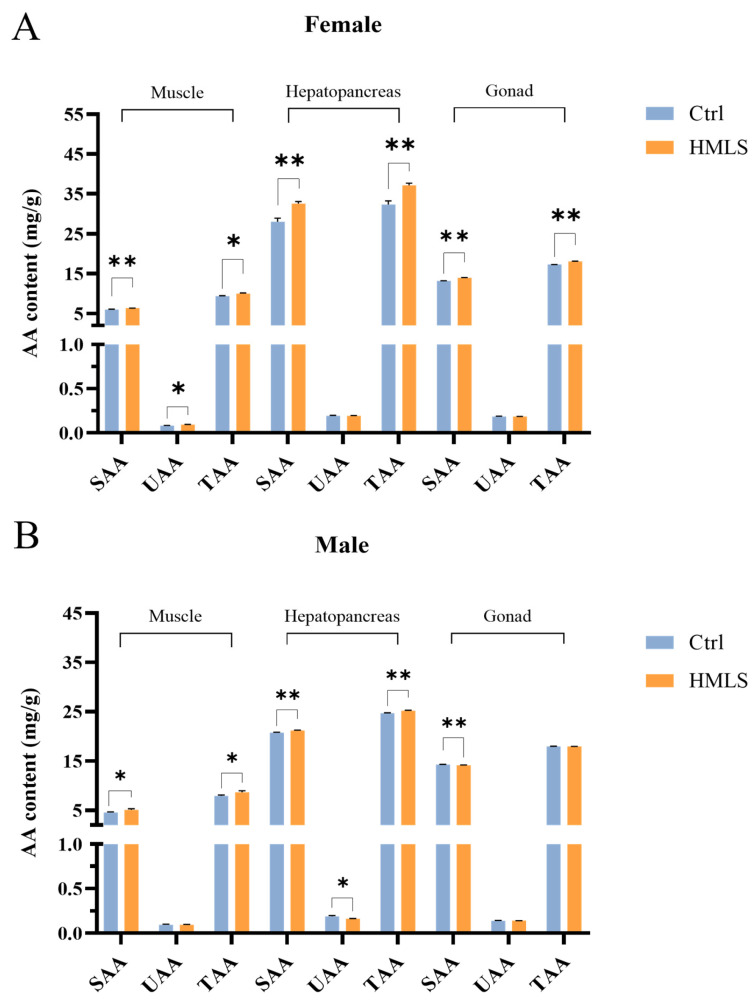
Effects of dietary HML on AA content of edible tissues in adult *E. sinensis*. (**A**) AA content of edible tissues in female *E. sinensis*. (**B**) AA content of edible tissues in male *E. sinensis*. The results are presented as the means ± SE (n = 9). An asterisk (*) indicates significance at the 0.05 level and ** at the 0.01 level.

The composition of delicious amino acids (DAAs) in edible tissues contributes to the umami taste and sweetness of crabs. To investigate the effects of dietary HML on the free AA composition in different edible tissues of steamed *E. sinensis*, a total of seventeen AAs were quantified, including six sweet amino acids (SAAs), two UAAs, and bitter amino acids (BAAs) (Table 2, Table 3 and Table 4). The total free amino acids (TAAs) content was highest in the hepatopancreas, followed by the gonad and muscle of both sexes (Figure 4A,B). Dietary HML significantly increased the SAA and TAA content in edible tissues, as well as the UAA content in the female muscle (*p* < 0.05). In male crabs, dietary HML significantly increased the SAA and TAA content in the muscle and hepatopancreas but significantly decreased the UAA content in the hepatopancreas and the SAA content in the gonad (*p* < 0.05).

As shown in Table 2, Table 3 and Table 4, glycine (Gly) was the dominant SAA in the edible tissues of crab. Dietary HML significantly increased the Gly content in the edible tissues of both sexes, except for the male gonad (*p* < 0.05). Furthermore, dietary HML significantly increased the threonine (Thr) content in the male edible tissues and female muscle, while significantly reducing the Thr content in the female gonad (*p* < 0.05). Compared with the Ctrl group, the proline (Pro) content in the gonad of both sexes, female muscle, and male hepatopancreas was significantly increased in the HMLS group (*p* < 0.05). Glutamic acid (Glu) was the dominant UAA in the edible tissues. Compared with the Ctrl group, the levels of Glu were significantly increased in the female muscle in the HMLS group but significantly decreased in the male hepatopancreas (*p* < 0.05).

The levels of BAA were also analyzed in the edible tissues of crabs. No significant differences were observed in the content of cysteine (Cys), methionine (Met), isoleucine (Ile), leucine (Leu), lysine (Lys), and histidine (His) between the Ctrl and HMLS groups (*p* > 0.05). However, the levels of valine (Val) were significantly increased in the edible tissues of crabs in the HMLS group compared to the Ctrl group. Dietary HML also significantly increased the levels of tyrosine (Tyr) in the male hepatopancreas, as well as in the female muscle and gonad. Furthermore, dietary HML significantly increased the levels of phenylalanine (Phe) in the male hepatopancreas and female gonad but significantly decreased the levels in the male muscle (*p* < 0.05).

### 3.6. Umami Nucleotide Content in Different Edible Tissues of Steamed E. sinensis

**Figure 5:**
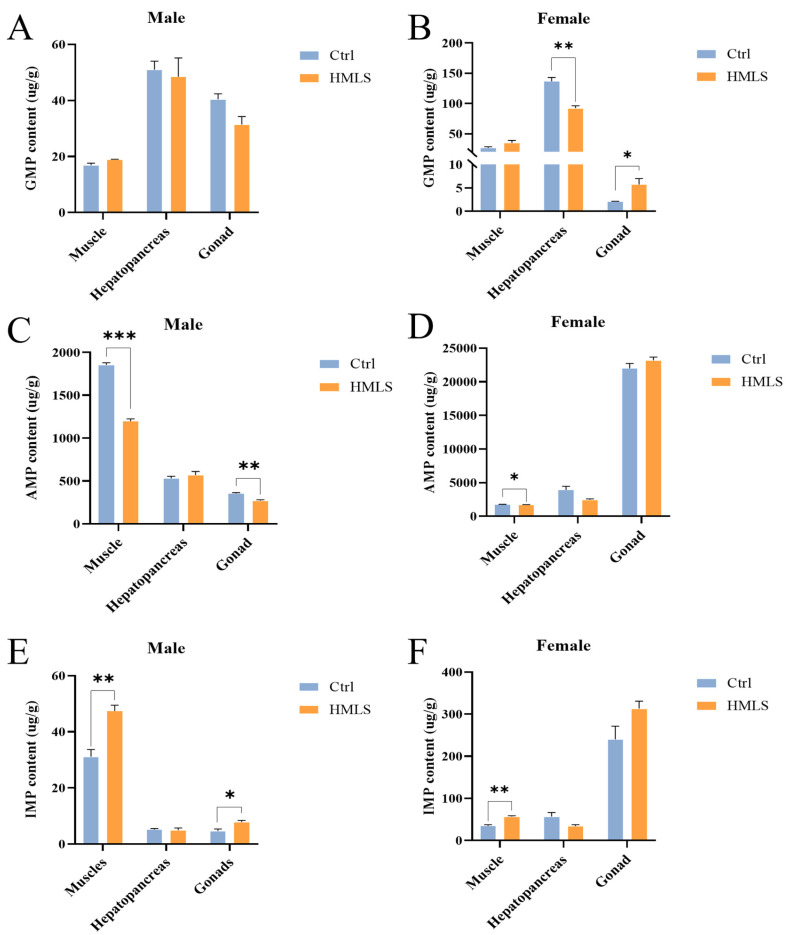
Effects of dietary HML on umami nucleotide content of edible tissues in adult *E. sinensis*. (**A**,**B**) GMP content. (**C**,**D**) AMP content. (**E**,**F**) IMP content. The results are presented as the means ± SE (n = 9). An asterisk (*) indicates significance at the 0.05 level, ** at the 0.01 level, and *** at the 0.001 level.

Umami nucleotides, including IMP, AMP, and GMP, were measured in various edible tissues of steamed crabs. As shown in Figure 5A, no significant differences were observed in the GMP content of the male edible tissues between the Ctrl group and HMLS group (*p* > 0.05). However, the GMP content of the female hepatopancreas in the Ctrl group was significantly higher than that in the HMLS group (*p* < 0.01) (Figure 5B). Dietary HML significantly decreased the AMP content in the muscle (*p* < 0.0001) and gonad (*p* < 0.01) of male crabs (Figure 5C), and in the muscle of female crabs (*p* < 0.05) (Figure 5D). In contrast, dietary HML significantly increased the IMP content in the muscle (*p* < 0.001) and gonad (*p* < 0.05) of male crabs (Figure 5E), as well as in the muscle of female crabs (*p* < 0.01) (Figure 5F). Furthermore, no significant differences were found in the AMP and IMP content in other edible tissues between the Ctrl group and HMLS group (*p* > 0.05) (Figure 5C–F).

### 3.7. Flavor Evaluation Indices in Different Edible Tissues of Steamed E. sinensis

The TAV > 1 was employed to assess the flavor characteristics in the edible tissues of steamed *E. sinensis*. The results indicated that Gly was the primary AA contributing to the sweetness of the edible tissues, while Val was the major AA responsible for bitterness. The highest TAV of Gly was observed in the female hepatopancreas. Additionally, nucleotide TAV analysis revealed that the TAV of AMP exceeded 1.0 only in the female gonad (Table 5, Table 6 and Table 7).

The SWT was utilized to quantify the sweetness imparted by SAAs in these tissues. As shown in Table 8, Table 9 and Table 10, the highest SWT was recorded in the female hepatopancreas, while the lowest SWT was found in the male muscle of both the Ctrl and HMLS groups. Following HML supplementation, SWT in the muscle and hepatopancreas of both sexes was significantly increased, whereas SWT in the male gonad was significantly decreased. These results indicated that dietary HML could significantly enhance the sweetness of the muscle and hepatopancreas.

EUC is an indicator of umami taste to evaluate the synergistic effects of UAAs and umami nucleotides. The strongest umami taste was observed in the female gonad. In contrast, the EUCs of the male muscle and gonad were significantly decreased in the HMLS group compared with the Ctrl group. Furthermore, dietary HML significantly decreased Table 4.

### 3.8. Radar Plots and Correlation Analysis

**Figure 6:**
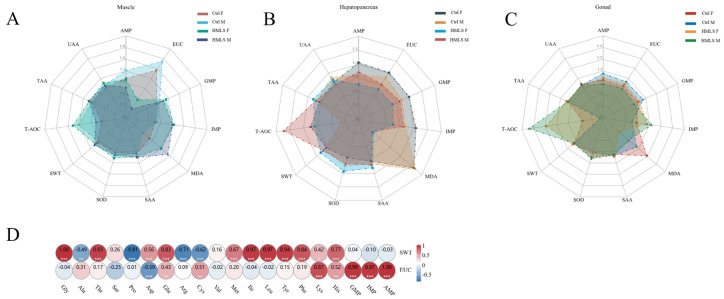
Radar plots and correlation analysis. (**A**–**C**) Radar plot of biomarker data of edible tissues (muscle, hepatopancreas, and gonad) in adult *E. sinensis*. (**D**) Correlation map of FAA/umami nucleotide content and flavor evaluation indices. The color scale indicates the correlation value, where vivid red indicates positive correlation, and vivid blue indicates negative correlation. (“*”: *p* < 0.05; “**”: *p* < 0.01; “***”: *p* < 0.001).

The radar plots indicated that the EUC in the muscle of the HMLS group was significantly lower than that of the Ctrl group (*p* < 0.05), whereas the levels of IMP and MDA in the muscle of the HMLS group were notably higher (*p* < 0.05) (Figure 6A). Compared with the Ctrl group, the MDA value in the hepatopancreas of the HMLS group was the lowest (*p* < 0.05), and the T-AOC value in the male hepatopancreas of the HMLS group was the highest (*p* < 0.05). Furthermore, the values of AMP, IMP, GMP, and EUC were significantly lower in the HMLS F group compared to the Ctrl F group (*p* < 0.05) (Figure 6B). In the gonad, the MDA value of the HMLS group was significantly lower than that of the Ctrl group (*p* < 0.05), while the IMP and T-AOC values in the gonad of the HMLS group were significantly higher (*p* < 0.05) (Figure 6C).

In Pearson’s correlation analysis, a correlation coefficient absolute value between 0.8 and 1.0 was considered to represent a strong correlation (Figure 6D). The SWT was positively correlated with Gly, Thr, Asp, Glu, Met, Ile, Leu, Tyr, Phe, Lys, and His, while it was negatively correlated with Ala, Pro, Arg, and Cys (*p* > 0.05). The EUC was positively correlated with Glu, Lys, His, Cys, GMP, IMP, and AMP, while it was negatively correlated with Asp (*p* > 0.05).

## 4. Discussion

Currently, the demand for high-quality *E. sinensis* is increasing, and a key factor in improving quality is optimizing the fattening process [9]. Diet composition and water environmental conditions have been identified as contributing factors to the final fattening outcomes in various studies [33]. The total edible parts of *E. sinensis* include meat, the hepatopancreas, and gonads [34]. A previous study reported that black soldier fly larvae can enhance the flavor profile of edible tissues in *E. sinensis* when used to replace traditional iced trash fish in the diet [8]. This suggests that insect-based diets may offer a viable approach to improving the nutritional value of edible parts. The use of HML has gained popularity in fish aquaculture in recent years [14,21]; however, limited research has focused on the effects of dietary HML on *E. sinensis* culture. In this study, we investigated the effects of a diet supplemented with HML on growth performance, antioxidant activity, and nutritional quality in adult *E. sinensis* following 40 days of fattening.

Previous studies have reported on the yield of edible tissues in *E. sinensis* during the fattening period [2,6,35]. In this study, the TEY following dietary HML supplementation was composed of MY (male: 27.23%; female: 24.81%), HSI (male: 8.78%; female: 8.85%), and GSI (male: 3.44%; female: 10.77%), which was consistent with previous findings. Efficient nutrient feeding is crucial for achieving optimal fattening effects in *E. sinensis*. The existing research has shown no significant differences in the HSI and GSI levels of *E. sinensis* fed FDs versus IF [34]. In comparison with formulated diets, dietary supplementation with astaxanthin or *Haematococcus pluvialis* had no significant effects on the HSI and GSI [35,36]. Additionally, it was also found that dietary supplementation with 0.4% cholesterol significantly upregulated both the HSI and GSI levels but had no significant effects on the MY in *E. sinensis* [37,38]. Furthermore, supplementation with 0.33% docosahexaenoic acid (DHA) oil significantly increased the GSI in *E. sinensis* [39]. Our results suggest that HML supplementation in formulated diets effectively improved the HSI of *E. sinensis*. Similar to our results, higher HSI values were observed in *O. niloticus* fed with diets containing HM [40]. Studies have shown that HML extract reduces lipid accumulation in the hepatopancreas of rats by modulating the peroxisome proliferator-activated receptor gamma (PPARγ) [41]; however, the specific effect of HML on higher HSI values of *E. sinensis* warrants further investigation.

Astaxanthin, a carotenoid compound, offers various health benefits for crustaceans [9,42]. Currently, astaxanthin is widely used in *E. sinensis* culture to enhance immune function, antioxidant capacity, and coloration [43,44]. Our findings demonstrated that HML feeding significantly increased the astaxanthin content in the carapaces of both male and female *E. sinensis*, as well as in the female hepatopancreas and gonad. Previous studies have shown that diets incorporating insect meals (such as cricket, grasshopper, and mealworm) can elevate carotenoid levels in *Xiphophorus maculatus* (Gunther, 1866) [45]. However, there is limited research on the effects of dietary HML on the astaxanthin content in *E. sinensis*, and the underlying mechanisms warrant further investigation.

It has been demonstrated that HML exhibit significant antioxidant potential when incorporated into the diets of hybrid catfish (*Clarias gariepinus* ♀ x *Heterobranchus longifilis* ♂) and (*Lithobates catesbeiana*) [14,46]. In contrast, the antioxidant capacity of prawn *Palaemon adspersus* was unaffected by the inclusion of HML in the diets (Rathke, 1837) [47]. HM have shown considerable promise in enhancing the antioxidant capacity of crustaceans by providing essential nutrients such as AAs, microelements, and chitin [11,12,13]. However, the effects of dietary HML on the antioxidant capacity of different edible tissues in *E. sinensis* remain underexplored. In the present study, we found that dietary HML enhanced the antioxidant capacity of the hepatopancreas and gonad in both male and female *E. sinensis*, as well as the female muscle.

Previous studies have demonstrated that a dietary insect meal has the potential to affect textural characteristics by altering the muscle fiber diameter and density [48]. Research has indicated that the functional components of black soldier fly (*Hermetia illucens*) larvae can improve the chewiness, cohesiveness, gumminess, and hardness of barramundi (*Lates calcarifer*) muscle [49]. Additionally, the inclusion of yellow mealworm (*Tenebrio molitor*) in the diet has been shown to affect muscle fiber density, which is positively correlated with muscle hardness, adhesiveness, springiness, chewiness, and gumminess [48]. Here, we observed that HML feeding enhanced the adhesiveness in the male muscle, while improving the cohesiveness, chewiness, and resilience in the female muscle. These changes can be attributed to alterations in muscle cellularity. However, the precise mechanisms by which HML-supplemented formula diets regulate muscle structure warrant further investigation.

Proteins are crucial energy sources for crustaceans, and AA composition serves as an important indicator for evaluating the nutritional value of *E. sinensis* [10]. HML are rich in AAs and are considered a more environmentally sustainable alternative to traditional protein sources such as soybean meal or fishmeal [17]. In comparison to the AA composition of soybean meal and fishmeal [14], HML in this study are particularly abundant in Leu, His, Ala, Phe, Asp, Tyr, and Cys. To date, there are limited studies exploring the effects of dietary HML on the AA composition of *E. sinensis*. However, research on other insect meals has reported that *H. illucens* is an efficient source of animal protein for crustaceans [50,51]. The substitution of IF by *H. illucens* has been shown to significantly increase the TAA content in the gonad and the DAA (SAAs and UAAs) content in the muscle and gonad of male *E. sinensis,* while significantly decreasing the TAA and DAA content in the hepatopancreas of female *E. sinensis* [8]. In contrast, our study found that dietary HML significantly increased the TAA and SAA content in the edible tissues of female *E. sinensis* and in the muscle and hepatopancreas of male *E. sinensis* but had no significant effects on the TAA content in the gonad of male *E. sinensis.* Furthermore, varying levels of dietary defatted superworm (*Zophobas atratus*) or *H. illucens* larvae as alternative protein sources did not affect the TAA content in juvenile Pacific white shrimp (*Penaeus vannamei*) [52,53]. Therefore, the effects of dietary HML on the FAA content in *E. sinensis* may be gender- and tissue-specific.

IMP serves as a taste enhancer in food, boosting sweetness while masking fishy, sour, bitter, and salty flavors [54]. A previous study demonstrated that dietary *H. illucens* significantly increases the IMP content in the muscle of both sexes and in the gonad of female *E. sinensis* [8]. Consistent with this finding, our study showed that dietary HML significantly increased the IMP content in the muscle of both sexes and in the gonads of male *E. sinensis*. However, dietary HML significantly decreased the AMP content in the muscle of both sexes and in the gonad of the male, as well as the GMP content in the hepatopancreas of the female. A previous study indicates that IMP and GMP are umami-tasting compounds commonly used as flavor enhancers, while AMP can be converted into IMP by the action of AMP deaminase [55]. Therefore, our results suggest that dietary HML in *E. sinensis* promote the conversion of AMP to IMP in the muscle of both sexes and the gonad of the male. Furthermore, proteins in the feed affect the composition of AAs and nucleotides in *E. sinensis*, which contribute to both the nutritive value and unique flavor [3,56,57]. Previous studies have highlighted that TAV serves as an indicator for evaluating the taste impacts of AAs and nucleotides on the flavor of *E. sinensis* [31,58]. The umami taste of IMP and GMP is considered stronger than that of glutamic acid (Glu) [54]. In the present study, the TAVs of UAAs, such as Glu and Asp, in the edible tissues of both the Ctrl and HMLS groups were below 1.0, with AMP primarily contributing to the umami taste in the female gonads (TAV greater than 1.0). Contrary to the results observed in *E. sinensis* fed *H. illucens* [8], IMP may be the primary contributor to the umami flavor in *E. sinensis*. Moreover, dietary HML for *E. sinensis* significantly decreased the EUC in the muscle and hepatopancreas of both sexes, as well as in the gonad of the male.

Previous studies have shown that certain SAAs, consisting of arginine (Arg), Ala, glycine (Gly), proline (Pro), and threonine (Thr), can have effects on the sweetness characteristics of edible tissues in *E. sinensis* [8]. However, limited research has focused on the impact of dietary HML on the sweetness or bitterness of edible tissues in aquatic species. Gly, for example, is known to provide a fragrant sweetness, reduce bitterness, and eliminate off-flavors in shrimp and crab [54]. In our study, the TAV of Gly was found to exceed 1.0 in all edible tissues. After HML feeding, the SWT of the muscle, hepatopancreas, and female gonad increased significantly, while the SWT in the male gonad decreased significantly. Similar changes in the SWT were observed in the muscle and male gonad of *E. sinensis* after feeding with *H. illucens* [8]. Additionally, valine (Val) is typically considered a bitter amino acid, characterized by weak bitterness and a subtle sweet taste [59]. A recent study suggested that a reduction in L-valine content in the muscle of *Megalobrama amblycephala* was significantly associated with increased sweetness [60]. However, in this study, the TAV of Val was greater than 1.0 across all edible tissues and showed a significant increase after HML feeding. Correlation analysis revealed no significant association between the Val content and either the EUC or SWT, suggesting that the increased Val content following HML feeding did not contribute to the sweetness in the edible tissues of *E. sinensis*.

## 5. Conclusions

In conclusion, this study demonstrated that dietary supplementation with HML significantly improved the HSI, astaxanthin content in female edible tissues, and flesh quality of *E. sinensis*. Moreover, HML feeding induced tissue- and sex-specific variations in the flavor characteristics. Specifically, dietary HML significantly reduced the EUC of the muscle, hepatopancreas, and male gonad, while significantly increasing the SWT of the muscle, hepatopancreas, and female gonad. Based on the current results, we have demonstrated that supplementation with HML is not only harmless to *E. sinensis* fattening but also significantly enhances the flavor characteristics of female crabs. Moving forward, we plan to focus on female crabs as the research subject, designing a multi-gradient HML supplementation to study its effects on edible tissues, and explore the balance between the growth of edible parts and the retention of umami.

## Figures and Tables

**Table 1:** Effects of dietary HMLS on textural properties in muscle.

Index	Female		Male	
	Ctrl	HMLS	Ctrl	HMLS
Hardness (g)	625.51 ± 90.68	805.38 ± 74.17	823.37 ± 73.21	1033.64 ± 74.39
Adhesiveness (J/m^3^)	6.54 ± 0.48	8.13 ± 1.33	8.22 ± 0.71	13.71 ± 1.16 **
Springiness (mm)	0.78 ± 0.03	0.84 ± 0.07	0.76 ± 0.04	0.77 ± 0.02
Cohesiveness (N)	0.56 ± 0.02	0.63 ± 0.01 **	0.60 ± 0.01	0.60 ± 0.011
Gumminess (N)	358.75 ± 36.79	502.91 ± 58.26	495.74 ± 39.78	617.70 ± 46.98
Chewiness (mJ)	278.89 ± 26.38	414.46 ± 40.59 *	384.40 ± 31.27	481.07 ± 42.84
Resilience (N)	0.27 ± 0.01	0.31 ± 0.01 **	0.30 ± 0.01	0.30 ± 0.01

Note. The results are presented as the means ± SE (n = 9). An asterisk (*) indicates significance at the 0.05 level, ** at the 0.01 level.

**Table 2:** Effects of dietary HMLS on amino acids content in muscle of steamed *E. sinensis* (mg/g wet weight).

AAs	Muscle			
	Female		Male	
	Ctrl	HMLS	Ctrl	HMLS
Gly	4.63 ± 0.10	4.94 ± 0.03 **	3.39 ± 0.18	3.88 ± 0.16 *
Ala	0.02 ± 0.00	0.02 ± 0.00	0.02 ± 0.00	0.02 ± 0.00
Thr	0.25 ± 0.00	0.26 ± 0.00 *	0.19 ± 0.01	0.27 ± 0.04 *
Ser	0.02 ± 0.00	0.02 ± 0.00	0.02 ± 0.00	0.02 ± 0.00
Pro	1.07 ± 0.00	1.11 ± 0.01 **	0.91 ± 0.03	0.91 ± 0.03
Arg	0.32 ± 0.01	0.49 ± 0.23	0.31 ± 0.01	0.32 ± 0.01
SAA	6.30 ± 0.10	6.83 ± 0.27 *	4.85 ± 0.16	5.41 ± 0.22 *
Asp	0.00 ± 0.00	0.00 ± 0.00	0.01 ± 0.00	0.01 ± 0.00
Glu	0.08 ± 0.00	0.09 ± 0.00 *	0.09 ± 0.01	0.09 ± 0.01
UAA	0.08 ± 0.00	0.09 ± 0.00 *	0.09 ± 0.01	0.09 ± 0.00
Cys	0.05 ± 0.00	0.05 ± 0.00	0.04 ± 0.00	0.04 ± 0.00
Val	1.69 ± 0.01	1.74 ± 0.01 **	1.93 ± 0.05	2.29 ± 0.09 **
Met	0.15 ± 0.00	0.15 ± 0.00	0.10 ± 0.01	0.10 ± 0.01
Ile	0.12 ± 0.00	0.12 ± 0.00	0.10 ± 0.00	0.10 ± 0.00
Leu	0.19 ± 0.01	0.19 ± 0.01	0.17 ± 0.00	0.17 ± 0.00
Tyr	0.32 ± 0.01	0.35 ± 0.01 *	0.28 ± 0.01	0.27 ± 0.01
Phe	0.34 ± 0.01	0.34 ± 0.01	0.28 ± 0.03 **	0.08 ± 0.02
Lys	0.09 ± 0.00	0.09 ± 0.00	0.08 ± 0.00	0.08 ± 0.01
His	0.03 ± 0.00	0.03 ± 0.00	0.03 ± 0.00	0.03 ± 0.00
TAA	9.37 ± 0.11	9.99 ± 0.26 *	7.93 ± 0.19	8.67 ± 0.27 *

Note. The results are presented as the means ± SE (n = 9). An asterisk (*) indicates significance at the 0.05 level, ** at the 0.01 level.

**Table 3:** Effects of dietary HMLS on amino acids content in hepatopancreas of steamed *E. sinensis* (mg/g wet weight).

AAs	Hepatopancreas			
	Female		Male	
	Ctrl	HMLS	Ctrl	HMLS
Gly	26.79 ± 0.92	31.37 ± 0.86 **	19.70 ± 0.08	20.07 ± 0.06 **
Ala	0.02 ± 0.00	0.02 ± 0.00	0.02 ± 0.00	0.01 ± 0.00
Thr	0.46 ± 0.02	0.46 ± 0.02	0.37 ± 0.00	0.39 ± 0.00 **
Ser	0.02 ± 0.00	0.02 ± 0.00	0.02 ± 0.00	0.02 ± 0.00
Pro	0.69 ± 0.00	0.69 ± 0.00	0.64 ± 0.00	0.68 ± 0.01 **
Arg	0.15 ± 0.00	0.16 ± 0.00	0.14 ± 0.00	0.14 ± 0.00
SAA	28.13 ± 0.90	32.71 ± 0.87 **	20.89 ± 0.09	21.31 ± 0.06 **
Asp	0.01 ± 0.00	0.01 ± 0.00	0.01 ± 0.00	0.01 ± 0.00
Glu	0.19 ± 0.01	0.19 ± 0.00	0.18 ± 0.01 **	0.16 ± 0.00
UAA	0.19 ± 0.01	0.20 ± 0.00	0.19 ± 0.01 **	0.16 ± 0.00
Cys	0.04 ± 0.00	0.04 ± 0.00	0.03 ± 0.00	0.03 ± 0.00
Val	1.96 ± 0.05	2.14 ± 0.06 *	1.74 ± 0.01	1.80 ± 0.01 **
Met	0.16 ± 0.00	0.16 ± 0.00	0.16 ± 0.00	0.16 ± 0.00
Ile	0.21 ± 0.01	0.21 ± 0.01	0.18 ± 0.00	0.18 ± 0.00
Leu	0.36 ± 0.01	0.36 ± 0.01	0.33 ± 0.01	0.33 ± 0.00
Tyr	0.51 ± 0.02	0.58 ± 0.05	0.47 ± 0.01	0.50 ± 0.01 **
Phe	0.56 ± 0.02	0.56 ± 0.02	0.52 ± 0.00	0.54 ± 0.00 **
Lys	0.15 ± 0.00	0.15 ± 0.00	0.13 ± 0.00	0.13 ± 0.00
His	0.05 ± 0.00	0.05 ± 0.00	0.04 ± 0.00	0.04 ± 0.00
TAA	32.31 ± 0.92	37.15 ± 0.91 **	24.69 ± 0.08	25.19 ± 0.07 **

Note. The results are presented as the means ± SE (n = 9). An asterisk (*) indicates significance at the 0.05 level, ** at the 0.01 level.

**Table 4:** Effects of dietary HMLS on amino acids content in gonad of steamed *E. sinensis* (mg/g wet weight).

AAs	Gonad			
	Female		Male	
	Ctrl	HMLS	Ctrl	HMLS
Gly	11.96 ± 0.04	12.72 ± 0.08 **	13.17 ± 0.03 **	13.02 ± 0.03
Ala	0.02 ± 0.00	0.02 ± 0.00	0.02 ± 0.00	0.01 ± 0.00
Thr	0.37 ± 0.00 **	0.35 ± 0.00	0.30 ± 0.00	0.32 ± 0.00 *
Ser	0.02 ± 0.00	0.02 ± 0.00	0.02 ± 0.00	0.02 ± 0.00
Pro	0.81 ± 0.01	0.84 ± 0.01 **	0.76 ± 0.00	0.77 ± 0.00 *
Arg	0.28 ± 0.01	0.28 ± 0.00	0.23 ± 0.00	0.23 ± 0.00
SAA	13.46 ± 0.04	14.23 ± 0.08 **	14.50 ± 0.04 **	14.37 ± 0.03
Asp	0.00 ± 0.00	0.00 ± 0.00	0.01 ± 0.00	0.01 ± 0.00
Glu	0.18 ± 0.00	0.18 ± 0.00	0.13 ± 0.00	0.13 ± 0.00
UAA	0.19 ± 0.00	0.18 ± 0.00	0.14 ± 0.00	0.14 ± 0.00
Cys	0.05 ± 0.00	0.05 ± 0.00	0.04 ± 0.00	0.04 ± 0.00
Val	1.88 ± 0.01	1.91 ± 0.01 **	1.85 ± 0.00	1.96 ± 0.04 **
Met	0.15 ± 0.00	0.15 ± 0.00	0.13 ± 0.00	0.13 ± 0.00
Ile	0.14 ± 0.00	0.14 ± 0.00	0.14 ± 0.00	0.14 ± 0.00
Leu	0.25 ± 0.00	0.25 ± 0.00	0.25 ± 0.00	0.25 ± 0.00
Tyr	0.42 ± 0.01	0.44 ± 0.00 **	0.37 ± 0.00	0.37 ± 0.00
Phe	0.45 ± 0.00	0.46 ± 0.00 **	0.39 ± 0.00	0.39 ± 0.00
Lys	0.22 ± 0.00	0.22 ± 0.00	0.11 ± 0.00	0.11 ± 0.00
His	0.05 ± 0.00	0.04 ± 0.00	0.03 ± 0.00	0.04 ± 0.00
TAA	17.26 ± 0.03	18.10 ± 0.09 **	17.96 ± 0.05	17.95 ± 0.02

Note. The results are presented as the means ± SE (n = 9). An asterisk (*) indicates significance at the 0.05 level, ** at the 0.01 level.

**Table 5:** Effects of dietary HMLS on the TAVs of amino acids and nucleotides in muscle.

AAs	Taste Threshold (mg/mL)	Muscle			
		Female		Male	
		Ctrl	HMLS	Ctrl	HMLS
Gly	1.3	1.78 ± 0.04	1.90 ± 0.01 **	1.30 ± 0.07	1.49 ± 0.06 *
Ala	0.6	0.02 ± 0.00	0.02 ± 0.00	0.01 ± 0.00	0.01 ± 0.00
Thr	2.6	0.05 ± 0.00	0.05 ± 0.00	0.04 ± 0.00	0.05 ± 0.01 *
Ser	1.5	0.01 ± 0.00	0.01 ± 0.00	0.01 ± 0.00	0.01 ± 0.00
Pro	3	0.18 ± 0.00	0.19 ± 0.00 **	0.15 ± 0.01	0.15 ± 0.00
Arg	0.5	0.32 ± 0.01	0.49 ± 0.23	0.31 ± 0.01	0.32 ± 0.01
SAA	0.5	0.00 ± 0.00	0.00 ± 0.00	0.01 ± 0.00	0.00 ± 0.00
Asp	0.2	0.20 ± 0.01	0.23 ± 0.01 *	0.22 ± 0.02	0.22 ± 0.01
Glu	0.2	0.13 ± 0.00	0.14 ± 0.00	0.10 ± 0.01	0.11 ± 0.01
UAA	0.4	2.11 ± 0.02	2.17 ± 0.01 **	2.41 ± 0.06	2.86 ± 0.11 **
Cys	0.3	0.25 ± 0.00	0.25 ± 0.00	0.16 ± 0.01	0.16 ± 0.01
Val	0.9	0.07 ± 0.00	0.07 ± 0.00	0.05 ± 0.00	0.05 ± 0.00
Met	1.4	0.07 ± 0.00	0.07 ± 0.00	0.06 ± 0.00	0.06 ± 0.00
Ile	0.7	0.23 ± 0.01	0.25 ± 0.01 *	0.20 ± 0.01	0.20 ± 0.01
Leu	0.9	0.19 ± 0.00	0.19 ± 0.01	0.15 ± 0.01 ***	0.05 ± 0.01
Tyr	0.5	0.09 ± 0.00	0.09 ± 0.00	0.08 ± 0.00	0.08 ± 0.01
Phe	0.2	0.08 ± 0.00	0.08 ± 0.00	0.07 ± 0.01	0.07 ± 0.01
Lys	0.1	0.01 ± 0.00	0.01 ± 0.00	0.01 ± 0.00	0.01 ± 0.00
His	0.2	0.01 ± 0.00	0.01 ± 0.00	0.01 ± 0.00	0.01 ± 0.00
TAA	0.5	0.14 ± 0.00	0.14 ± 0.00	0.15 ± 0.00 ***	0.10 ± 0.00

The results are presented as the means ± SE (n = 9). An asterisk (*) indicates significance at the 0.05 level, ** at the 0.01 level, and *** at the 0.001 level.

**Table 6:** Effects of dietary HMLS on the TAVs of amino acids and nucleotides in hepatopancreas.

AAs	Taste Threshold (mg/mL)	Hepatopancreas			
		Female		Male	
		Ctrl	HMLS	Ctrl	HMLS
Gly	1.3	10.30 ± 0.36	12.06 ± 0.33 **	7.58 ± 0.03	7.72 ± 0.02 **
Ala	0.6	0.01 ± 0.00	0.01 ± 0.00	0.01 ± 0.00	0.01 ± 0.00
Thr	2.6	0.09 ± 0.00	0.09 ± 0.00	0.07 ± 0.00	0.08 ± 0.00 **
Ser	1.5	0.01 ± 0.00	0.01 ± 0.00	0.01 ± 0.00	0.01 ± 0.00
Pro	3	0.12 ± 0.00	0.12 ± 0.00	0.10 ± 0.00	0.11 ± 0.00 **
Arg	0.5	0.15 ± 0.00	0.16 ± 0.00	0.14 ± 0.00	0.14 ± 0.00
SAA	0.5	0.01 ± 0.00	0.01 ± 0.00	0.01 ± 0.00	0.01 ± 0.00
Asp	0.2	0.47 ± 0.02	0.47 ± 0.01	0.46 ± 0.02 **	0.39 ± 0.01
Glu	0.2	0.09 ± 0.01	0.09 ± 0.01	0.09 ± 0.00	0.09 ± 0.00
UAA	0.4	2.45 ± 0.06	2.68 ± 0.08 *	2.17 ± 0.01	2.25 ± 0.01 ***
Cys	0.3	0.27 ± 0.01	0.27 ± 0.01	0.27 ± 0.01	0.27 ± 0.00
Val	0.9	0.12 ± 0.00	0.12 ± 0.00	0.10 ± 0.00	0.10 ± 0.00
Met	1.4	0.13 ± 0.00	0.13 ± 0.00	0.12 ± 0.00	0.12 ± 0.00
Ile	0.7	0.36 ± 0.01	0.41 ± 0.03	0.33 ± 0.00	0.36 ± 0.01 **
Leu	0.9	0.31 ± 0.01	0.31 ± 0.01	0.29 ± 0.00	0.30 ± 0.00 **
Tyr	0.5	0.15 ± 0.00	0.15 ± 0.00	0.13 ± 0.00	0.13 ± 0.00
Phe	0.2	0.11 ± 0.01	0.12 ± 0.01	0.10 ± 0.00	0.10 ± 0.00
Lys	0.1	0.05 ± 0.00 **	0.04 ± 0.00	0.02 ± 0.00	0.02 ± 0.00
His	0.2	0.01 ± 0.00	0.01 ± 0.00	0.00 ± 0.00	0.00 ± 0.00
TAA	0.5	0.31 ± 0.07	0.20 ± 0.02	0.04 ± 0.00	0.05 ± 0.01

The results are presented as the means ± SE (n = 9). An asterisk (*) indicates significance at the 0.05 level, ** at the 0.01 level, and *** at the 0.001 level.

**Table 7:** Effects of dietary HMLS on the TAVs of amino acids and nucleotides in gonad.

AAs	Taste Threshold (mg/mL)	Gonad			
		Female		Male	
		Ctrl	HMLS	Ctrl	HMLS
Gly	1.3	4.60 ± 0.01	4.89 ± 0.03 **	5.06 ± 0.01 **	5.01 ± 0.01
Ala	0.6	0.02 ± 0.00	0.02 ± 0.00	0.01 ± 0.00	0.01 ± 0.00
Thr	2.6	0.07 ± 0.00	0.07 ± 0.00	0.06 ± 0.00	0.06 ± 0.00
Ser	1.5	0.01 ± 0.00	0.01 ± 0.00	0.01 ± 0.00	0.01 ± 0.00
Pro	3	0.13 ± 0.00	0.14 ± 0.00 **	0.12 ± 0.00	0.13 ± 0.00 *
Arg	0.5	0.28 ± 0.01	0.28 ± 0.00	0.23 ± 0.00	0.23 ± 0.00
SAA	0.5	0.00 ± 0.00	0.00 ± 0.00	0.01 ± 0.00	0.01 ± 0.00
Asp	0.2	0.45 ± 0.01	0.45 ± 0.01	0.34 ± 0.00	0.34 ± 0.00
Glu	0.2	0.13 ± 0.01	0.13 ± 0.01	0.10 ± 0.01	0.10 ± 0.00
UAA	0.4	2.35 ± 0.01	2.39 ± 0.01 **	2.31 ± 0.01	2.45 ± 0.05 **
Cys	0.3	0.26 ± 0.01	0.25 ± 0.00	0.22 ± 0.00	0.22 ± 0.00
Val	0.9	0.08 ± 0.00	0.08 ± 0.00	0.08 ± 0.00	0.08 ± 0.00
Met	1.4	0.09 ± 0.00	0.09 ± 0.00	0.09 ± 0.00	0.09 ± 0.00
Ile	0.7	0.30 ± 0.01	0.32 ± 0.00 **	0.27 ± 0.00	0.26 ± 0.00
Leu	0.9	0.25 ± 0.00	0.26 ± 0.00 **	0.22 ± 0.00	0.22 ± 0.00
Tyr	0.5	0.22 ± 0.00	0.22 ± 0.00	0.11 ± 0.00	0.11 ± 0.00
Phe	0.2	0.11 ± 0.01	0.11 ± 0.01	0.08 ± 0.00	0.09 ± 0.00
Lys	0.1	0.27 ± 0.01	0.28 ± 0.01	0.02 ± 0.00 *	0.01 ± 0.00
His	0.2	0.05 ± 0.01	0.06 ± 0.01	0.00 ± 0.00	0.00 ± 0.00
TAA	0.5	1.76 ± 0.10	1.86 ± 0.06	0.03 ± 0.00 **	0.02 ± 0.00

The results are presented as the means ± SE (n = 9). An asterisk (*) indicates significance at the 0.05 level, ** at the 0.01 level.

**Table 8:** Effects of dietary HMLS on the SWT and EUC in muscle.

AAs	Muscle			
	Female		Male	
	Ctrl	HMLS	Ctrl	HMLS
EUC	1.80 ± 0.08 ***	0.51 ± 0.04	2.11 ± 0.12 **	0.34 ± 0.02
SWT	0.37 ± 0.01	0.39 ± 0.00 **	0.27 ± 0.01	0.31 ± 0.01 *

The results are presented as the means ± SE (n = 9). An asterisk (*) indicates significance at the 0.05 level, ** at the 0.01 level, and *** at the 0.001 level.

**Table 9:** Effects of dietary HMLS on the SWT and EUC in hepatopancreas.

AAs	Hepatopancreas			
	Female		Male	
	Ctrl	HMLS	Ctrl	HMLS
EUC	2.47 ± 0.38 *	1.60 ± 0.12	0.51 ± 0.03 *	0.43 ± 0.03
SWT	2.14 ± 0.07	2.51 ± 0.07 **	1.58 ± 0.01	1.61 ± 0.00 **

The results are presented as the means ± SE (n = 9). An asterisk (*) indicates significance at the 0.05 level, ** at the 0.01 level.

**Table 10:** Effects of dietary HMLS on the SWT and EUC in gonad.

AAs	Gonad			
	Female		Male	
	Ctrl	HMLS	Ctrl	HMLS
EUC	12.77 ± 0.86	13.56 ± 0.51	0.28 ± 0.02 *	0.23 ± 0.02
SWT	0.95 ± 0.00	1.02 ± 0.01 **	1.05 ± 0.00 **	1.04 ± 0.00

The results are presented as the means ± SE (n = 9). An asterisk (*) indicates significance at the 0.05 level, ** at the 0.01 level.

## Data Availability

The original contributions presented in this study are included in the article/Supplementary Materials, and further inquiries can be directed to the corresponding author.

## Supplementary Materials

The following supporting information can be downloaded at: https://www.mdpi.com/article/10.3390/foods14071250/s1, Table S1: Nutritional composition of HML.
