# Supplementary material for: Fattening by Dietary Replacement with Fly Maggot Larvae (Musca domestica) Enhances the Edible Yield, Antioxidant Capability, Nutritional and Taste Quality of Adult Chinese Mitten Crab Eriocheir sinensis
Source: Foods. 2025 Apr 3;14(7):1250. doi: 10.3390/foods14071250 (PMC11989204; doi:10.3390/foods14071250)
Supplement: Supplementary file 1 [file foods-14-01250-s001.zip › foods-3532827-supplementary.pdf]

Supplementary Table S1

Nutritional composition of HML

**Proximate composition**	**Contents**	**Mineral**	**Contents**
Crude protein (%)	59.78	k（%)	1.22
Total lipid (%）	14.98	Na（mg/kg)	8211.46
Moisture (%）	18.22	Mg（%)	1.11
Crude ash (%)	10.12	Ca（%)	0.39
Crude fiber (%)	7.98	P（%）	2.29
**Amino acids (mg g^{-1} dry diets)**	**Contents**	Mn（mg/kg)	349.15
Asp	5.646	Zn（mg/kg)	331.04
Thr	2.046	Fe（mg/kg)	271.11
Ser	2.567	Se（mg/kg)	6.59
Glu	8.555	Cu（mg/kg)	18.23
Pro	1.989	Cr（mg/kg)	1.11
Gly	2.347	Cd（mg/kg)	0.19
Ala	3.333	Hg（mg/kg)	0.11
Cys	0.616	Pb（mg/kg)	0.23
Val	2.549	As（mg/kg)	0.09
Met	2.216	**Total energy (KJ/g)**	20.04
Ile	1.99		
Leu	3.67		
Tyr	4.142		
Phe	3.679		
Lys	3.971		
His	1.246		
Arg	2.589		
TAA（g/100g）	53.151		

HML:Housefly maggot larvae; TAA: total amino acids.
